# Subjective global assessment of malnutrition and dysphagia effect on the clinical and Para-clinical outcomes in elderly ischemic stroke patients: a community-based study

**DOI:** 10.1186/s12883-021-02501-4

**Published:** 2021-11-30

**Authors:** Mahsa Mahmoudinezhad, Mohammad Khalili, Nasim Rezaeemanesh, Mehdi Farhoudi, Sharareh Eskandarieh

**Affiliations:** 1 Student Research Committee, Tabriz University of Medical Sciences, Tabriz, Iran; 2 Multiple Sclerosis Research Center, Neuroscience Institute, Tehran University of Medical Sciences, Tehran, Iran; 3 Neurosciences Research Center, Tabriz University of Medical Sciences, Tabriz, Iran

**Keywords:** Stroke, mRS, NIHSS, Malnutrition

## Abstract

**Background:**

Malnutrition as a result of insufficient intake or uptake of nutrition leads to increasing rate of chronic diseases such as stroke. Stroke is one of the most common causes of death in western countries and its increasing trend has attracted lots of attention. In this regard, it seems logical to focus on modifiable risk factors such as nutrition, in order to reduce the resulting complications. Accordingly, this study aimed at evaluating nutrition status of stroke patients to estimate its relationship with clinical outcomes of stroke.

**Methods:**

In the present cross-sectional study, 349 patients were recruited. Nutrition assessment was performed using Patient-Generated Subjective Global Assessment (PG-SGA). In addition, National Institutes of Health Stroke Scale (NIHSS), modified Rankin Scale (mRS), and biochemical tests were performed.

**Results:**

Our findings elucidated a significant positive correlation of mRS with PG-SGA and consciousness score, as well as a negative correlation with BMI, calf circumference, mid-arm circumference, and triceps skinfold at admission time (*P* ≤ 0.002). Moreover, a direct correlation was found between mRS and PG-SGA and consciousness score at discharge time (*P* ≤ 0.001). In contrast, an inverse correlation was established between mRS and mid-arm circumference (*P* = 0.02). Furthermore, univariate analysis indicated significant associations between mRS ≥ 3 and age (OR: 1.02; 95%CI: 1.00–1.04), PG-SGA (OR: 1.08; 95%CI: 1.03–1.13), NIHSS (OR: 1.04; 95%CI: 1.02–1.07), dysphagia (OR: 1.69; 95%CI: 1.03–2.77), consciousness (OR: 1.48; 95%CI: 1.07–2.04), and mid-arm circumference (OR: 0.95; 95%CI: 0.90–1.00). In addition, these associations remained significant in multivariate analysis for PG-SGA (OR: 1.07; 95%CI: 1.00–1.13) and NIHSS (OR: 1.04; 95%CI: 1.01–1.07).

**Conclusion:**

This study revealed a positive correlation between mRS and consciousness status and PG-SGA score, as well as a negative one between mRS and MAC at discharge time.

## Background

Malnutrition as a result of insufficient intake or uptake of nutrition, leads to increased rate of some medical complications and diseases [1]. It can alter body composition and cell mass, decrease fat-free mass, and worsen medical outcomes of some diseases such as stroke [1]. Stroke is one of the most common causes of death in western countries and regarding its increasing trend, it is required to focus on modifiable risk factors to prevent its prevalence. Previous studies have demonstrated that (16–49)% of acute stroke patients suffered from malnutrition at admission time [2, 3]. In addition, malnutrition may increase the length of hospitalization, readmissions, healthcare costs, poor functional outcome, and mortality rate, as well as decrease life quality and survival [1].

Hence, there is a large body of data supporting the concept that nutrition evaluation in hospitalized stroke patients is critical and would give us a starting point to look at this issue as a priority [4, 5]. To this end, several tools have been developed to identify patients at risk of malnutrition and consider proper nutritional interventions. These tools are classified to objective and subjective evaluations. The objective one is based on the hematological and biochemical parameters. In this regard, the Patient-Generated Subjective Global Assessment (PG-SGA) is a widely used subjective nutritional screening tool including assessment of body weight, food intake, symptoms, relationship between disease and nutritional requirement, metabolism requirement, and physical examination [6, 7]. Accordingly, this study aimed to evaluate nutritional status of stroke patients at admission time and identified malnourished patients to estimate its relationship with clinical outcomes of stroke.

## Methods

### Study design and participants

The present cross-sectional study recruited patients referring to Imam Reza University Hospital of Tabriz (using Tabriz Stroke Registry database) and Firozgar and Sina hospitals of Tehran from April 2020 to November 2020. Physical examination, computed tomography (CT) scan, and magnetic resonance imaging (MRI; for further investigations) were performed on all patients in the first 24 h of their admission and those who met the inclusion criteria including: patients aged 65 years and older, definite stroke diagnosis confirmation by a neurologist, hospitalization over than 48 h. However, patients with a history of stroke with dysphagia, head/neck surgery, or trauma affecting swallowing ability were excluded from the study. Patients with treatment of tissue plasminogen activator were excluded too. Written informed consent was obtained from all the participants at the beginning of the study. The protocol of the study was approved by the ethics committee of Tabriz University of Medical Sciences (No. TBZMED.REC.1394.507). All methods of study were conducted according to **Declarations of Helsinki and** funded by National Institute of Medical Research Development (NIMAD) with project no. 987875.

### Demographic and anthropometric assessment

In term of anthropometric assessments, the knee height of patients was measured with a non-stretchable tape to estimate height value at admission as follow:

64.19- (0.04× age) + (2.02× knee height) for men and 84.88 – (0.24 × age) + (1.83 × knee height) for women. A brief questionnaire containing demographic data and comorbidities such as diabetes, cardiovascular, arthritis, renal, gastrointestinal, thyroid, and liver diseases was completed for every patient. Also, demographic data in relation to medication intake and history of dysphagia were gathered through interview and medical record. Anthropometric data gathering, PG-SGA, modified Rankin Scale (mRS), dysphagia assessment, and biochemical tests were performed within 12 to 24 h after admission.

Anthropometric measurements, including body weight, height, body mass index (BMI), calf circumference (CC), mid-arm circumference (MAC), and triceps skinfold (TSF) thickness were done according to standards.

### Biochemical assessment

Serum glucose, total cholesterol (TC), triglyceride (TG), high-density lipoprotein cholesterol (HDL-C), albumin, high-sensitivity C-reactive protein (hs-CRP), and erythrocyte sedimentation rate (ESR) were measured within 12 to 24 h of stroke onset. In samples with TG < 400 mg/dL, low-density lipoprotein cholesterol (LDL-C) levels were calculated using the Friedewald equation: LDL-C (mg/dl)¼ TC_HDL-C_TG/5.

### Nutrition assessment

Nutrition assessments were done within 12 to 24 h after admission time, using PG-SGA, which includes: weight, dietary intake, activity level, relationship between disease and nutritional requirement, metabolism requirement, and physical examination. Total score was obtained by summing the scores of each item. Consequently, the score 0–1 indicated that no intervention was required, 2–3 meant education was needed by a trained one, 4–8 suggested intervention was required, and 9 indicated that intensive intervention was required. Finally, the patients were assigned into one of the 3 groups: well nourished, moderately malnourished, and severely malnourished.

### Dysphagia assessment

Dysphagia assessment was done within 12 to 24 h after admission. Due to impaired swallowing, dysphagia evaluation was done using the Northwestern Dysphagia Patient Check Sheet (NDPCS) [8]. The NDPCS included 28 items in 5 categories: medical history (4 items), behavioral variables (6 items), gross motor function (2 items), promotor test (9 items), and observations during swallowing (7 items). Each item was scored “safe” or “unsafe”. At last, the final score was obtained from the total number of “unsafe” items. To evaluate the swallowing ability, patients were requested to drink 1 mL thin liquid, 1 mL pudding, and a quarter of a Lorna Doone cookie (if chewing was possible).

### Stroke severity

Stroke related disability was assessed using modified Rankin Scale (mRS) by a trained one at admission and discharge times. It consists of 7 grades from 0 (no symptom) to 6 (death) as below: score 1 indicates no significant disability and the patient is able to do every usual activity. Score 2 shows a little disability and the patient is able to do part of his/her personal tasks without any assistance. Patients with score 3 have a moderate disability but they can walk without assistance. Score 4 is assigned to a group of patients with moderately severe disability who need support for walking. Patients with score 5 are bed ridden and require nursing care. Moreover, the severity of stroke in patients was assessed using National Institutes of Health Stroke Scale (NIHSS).

### Statistical analyses

Data were analyzed using SPSS 25.0 software. Normally distributed variables were reported by mean ± SD and nonparametric variables were reported as median and interquartile range. One-way analysis of variance (ANOVA) and Kruskal–Wallis tests were applied for comparison between groups. The *P*-value < 0.05 was considered as the significant level in all statistical analyses.

## Results

Baseline characteristics of 349 stroke patients at admission time are presented in Table 1. Study participants had the mean age of 70.91 ± 11.07 years and 186 (53.4%) of them were male. The main percent of patients lived with their spouses (207 (63.3%)) or other family members (83 (25.4%)) and 304 (87.9%) of participants were married. The majority of patients were illiterate (202 (61.0%)) and only 13.8% of them were unemployed. 257 (76.9%) subjects were non-smokers. Hypertension was the most prevalent medical condition (69.0%) followed by cardiovascular diseases (31.5%), diabetes (26.9%), and hyperlipidemia (25.3%). The results of CT scan revealed that 94.0% of strokes were ischemic and the others were hemorrhagic.

**Table 1:** Demographic, clinical and biochemical characteristics of participants at the admission time to hospital

Variables	n% OR Mean ± SD
**Age (year)**	70.91 ± 11.07
**Gender**	
Female	162 (46.6%)
Male	186 (53.4%)
**Living status**	
Living with spouse	207 (63.3%)
Living alone	34 (10.4%)
Living with family member other than spouse	83 (25.4%)
Living with nurse	1 (0.3%)
**Marital status**	
Single	10 (2.9%)
Married	304 (87.9%)
**Educational level**	
Illiterate	202 (61.0%)
Primordial/ Guidance	69 (20.8%)
Diploma	30 (9.1%)
Bachelor or higher	24 (7.3%)
**Occupation**	
Unemployed	44 (13.8%)
Employee	5 (1.6%)
Self-employment	64 (20.0%)
Retired	65 (20.3%)
Other	142 (44.4%)
**Smoking**	
No	257 (76.9%)
Yes	77 (23.1%)
**Medical history**	
Hypertension	236 (69.0%)
Hyperlipidemia	88 (25.3%)
Diabetes	94 (26.9%)
Cardiovascular diseases	110 (31.5%)
**CT Scan**	
Ischemic	327 (94.0%)
Hemorrhagic	20 (5.7%)
**Dysphagia**	
No	210 (60.5%)
Yes	136 (39.2%)
**NIHSS**	15.85 ± 12.27
**mRS score**	4.14 ± 1.69
**mRS status**	
≤ 2	74 (21.4%)
≥ 3	272 (78.6%)
**Consciousness**	1.65 ± 0.79
**Albumin (g/dl)**	3.55 ± 1.62
**TG (mg/dl)**	142.99 ± 75.10
**LDL-C (mg/dl)**	123.50 ± 64.95
**HDL-C (mg/dl)**	45.55 ± 10.56
**Hemoglobin (g/dl)**	12.99 ± 2.13
**Hematocrit**	39.60 ± 5.77

*NIHHS* National Institutes of Health Stroke Scale, *mRS* Modified Rankin Scale, *TG* Triglycerides, *HDL-C* High-density lipoprotein cholesterol, *LDL-C* Low-density lipoprotein cholesterol

The mean NIHSS score of participants was 15.85 ± 12.27. Among the patients,136 (39.2%) suffered from dysphagia. The mean mRS score of patients was 4.14 ± 1.69; 74 (21.4%) patients got an mRS score of less than 2, and 272 (78.6%) got a score more than 3. The mean score of patients’ consciousness status was 1.65 ± 0.79. The mean levels of biochemical parameters such as albumin, TG, LDL-C, HDL-C, hemoglobin, and hematocrit could be found in Table 1. The mean level of all parameters were within the normal range.

Table 2 highlights the anthropometric, nutritional, and feeding status of participants at the time of admission. The mean score of PG-SGA was 10.62 ± 5.24. Ten (2.9%) of study participants had a PG-SGA score between 0 and 1 and did not require any intervention. PG-SGA score of 22 (6.4%) patients was between 2 and 3, which suggested the necessity of the education of the patients and their family, and pharmacological intervention. Moreover, 71 (20.6%) of them required nutritional intervention due to PG-SGA score (i.e., 4–8). Others (242 (70.1%)) had a PG-SGA score ≥ 9, thus required nutrient intervention and/ or improved symptom management. Stroke patients had the mean BMI of 25.89 ± 4.62 kg/m^2^. The majority of patients (182 (56.2%)) received oral feeding and 30.2% (98 subjects) were under nasogastric intubation.

**Table 2:** Anthropometric, nutritional and feeding status of participants at the time of admission to hospital

Variables	n% OR Mean ± SD
**PG-SGA score**	10.62 ± 5.24
**Nutritional status**	
No intervention required	10 (2.9%)
Patients and family education and pharmacological intervention	22 (6.4%)
Dietary intervention required	71 (20.6%)
Critical need for nutrient intervention and/ or improved symptom management	242 (70.1%)
**BMI (kg/m**^**2**^**)**	25.89 ± 4.62
**Feeding method in hospital**	
Oral	182 (56.2%)
Parenteral	37 (11.4%)
Nasogastric intubation	98 (30.2%)
Percutaneous endoscopic gastrostomy	6 (1.9%)
**Knee height (cm)**	47.74 ± 4.51
**Calf circumference (cm)**	33.71 ± 4.63
**Mid-arm circumference (cm)**	28.13 ± 5.14
**Triceps skinfold (cm)**	13.14 ± 6.67

*PG-SGA* Patient-generated Subjective Global Assessment

Table 3 compares demographic, clinical, biochemical, and anthropometric parameters among the PG-SGA status groups. Age, at admission mRS, at discharge mRS, NIHSS, consciousness score, and hemoglobin showed significant increasing trends based on the PG-SGA classification *(P* ≤ 0.002) while BMI, calf circumference, TG, and hematocrit indicated decreasing trends (*P* ≤ 0.02). Living status was significantly different between groups (*P*: 0.01), the most prevalent status in all groups was living with spouse. There were no differences in term of mid-arm circumference, triceps skinfold, dysphagia, albumin, LDL-C, and HDL-C between the four classifications of PG-SGA score (*P* > 0.05).

**Table 3:** Comparison of demographic, clinical, biochemical and anthropometric parameters among the PG-SGA status groups

Variables	Nutritional Status based on PG-SGA score^a^				***P*** value
	1	2	3	4	
**Age (year)**	65.70 ± 6.17	68.90 ± 10.19	68.89 ± 9.54	71.57 ± 11.67	< 0.001
**Living status**					0.01
Living with spouse	8 (80.0%)	14 (66.7%)	46 (69.7%)	138 (60.8%)	
Living alone	2 (20.0%)	4 (19.0%)	4 (6.1%)	24 (10.6%)	
Living with family member other than spouse	0 (0.0%)	2 (9.5%)	16 (24.2%)	63 (27.8%)	
Living with nurse	0 (0.0%)	1 (4.8%)	0 (0.0%)	0 (0.0%)	
**PG-SGA score**	0.80 ± 0.42	2.32 ± 0.48	6.00 ± 1.39	13.14 ± 3.96	< 0.001
**BMI (kg/m**^**2**^**)**	31.06 ± 3.76	26.07 ± 4.28	27.24 ± 3.83	25.52 ± 4.75	0.02
**Knee height (cm)**	45.33 ± 4.51	45.70 ± 4.19	46.77 ± 3.84	48.28 ± 4.63	0.50
**Calf circumference (cm)**	36.67 ± 2.31	34.42 ± 5.38	33.90 ± 5.08	33.61 ± 4.49	0.001
**Mid-arm circumference (cm)**	32.50 ± 2.18	27.93 ± 4.64	29.38 ± 3.74	27.82 ± 5.48	0.06
**Triceps skinfold (cm)**	19.67 ± 5.03	12.60 ± 7.18	10.94 ± 4.83	13.78 ± 6.97	0.25
**mRS admission**	2.30 ± 1.25	3.64 ± 1.68	3.94 ± 1.82	4.32 ± 1.60	< 0.001
**mRS follow-up**	2.70 ± 1.06	3.09 ± 1.02	3.17 ± 1.28	3.57 ± 1.58	< 0.001
**NIHSS**	11.10 ± 10.92	12.27 ± 10.00	14.37 ± 10.96	16.70 ± 12.78	< 0.001
**Dysphagia**					0.06
No	9 (90.0%)	16 (72.7%)	46 (65.7%)	135 (56.0%)	
Yes	1 (10.0%)	6 (27.3%)	23 (32.9%)	106 (44.0%)	
**Consciousness**	1.20 ± 0.42	1.32 ± 0.65	1.54 ± 0.76	1.74 ± 0.80	< 0.001
**Albumin (g/dl)**	2.90 ± 0.00	4.09 ± .65	3.70 ± 0.65	3.50 ± 1.78	0.10
**TG (mg/dl)**	180.86 ± 75.74	133.07 ± 68.37	137.24 ± 88.21	144.36 ± 72.35	0.007
**LDL (mg/dl)**	88.50 ± 42.67	133.07 ± 68.37	137.24 ± 88.21	144.36 ± 72.35	0.45
**HDL (mg/dl)**	39.71 ± 6.75	42.27 ± 9.74	42.29 ± 10.65	46.54 ± 10.58	0.25
**Hemoglobin (g/dl)**	12.51 ± 2.43	13.61 ± 2.27	13.21 ± 1.78	12.86 ± 2.19	0.002
**Hematocrit**	41.06 ± 3.63	41.27 ± 5.88	40.68 ± 4.87	39.03 ± 5.98	0.002

*PG-SGA* Patient-generated Subjective Global Assessment, *NIHHS* National Institutes of Health Stroke Scale, *mRS* Modified Rankin Scale, *TG* Triglycerides, *HDL-C* High-density lipoprotein cholesterol, *LDL-C* Low-density lipoprotein cholesterol

^a^PG-SGA score: 1 = No intervention required at this time, 2 = Patients and family education and pharmacological intervention are needed, 3 = Dietary intervention required, 4 = Critical need for nutrient intervention and/ or improved symptom management

Table 4 indicates the correlation between at admission and discharge mRS and PG-SGA score, consciousness, albumin, and anthropometric variables. At admission mRS indicated significant direct correlations with PG-SGA score (r: 0.33) and consciousness score (r: 0.33), while it showed inverse correlations with BMI (r: − 0.17), calf circumference (r: − 0.32), mid-arm circumference (r: − 0.17), and triceps skinfold (r: − 0.20) (*p* ≤ 0.002). At discharge time, the correlations between mRS and PG-SGA score (r: 0.27), and consciousness score (r: 0.24) were also direct and significant (*P* < 0.001) and the correlation between mRS and mid-arm circumference (r: − 0.12) was inverse and significant (*P*:0.02). However, there was no significant correlation between mRS and BMI (r: − 0.09), calf circumference (r: − 0.08), and triceps skinfold (r: − 0.03) (*p* > 0.05) at discharge time. Albumin showed a non-significant correlation with mRS in both admission (*P*:0.66; r:0.02) and discharge (P:0.90; r:0.00) times.

**Table 4:** The correlation between at admission and discharge mRS with PG-SGA score, Consciousness, Albumin and anthropometric measurements

Variables		PG-SGA score	Consciousness	Albumin (g/dl)	BMI (kg/m2)	Calf circumference (cm)	Mid-arm circumference (cm)	Triceps skinfold (cm)
At admission mRS	r ***P*** value	0.33 < 0.001	0.53 < 0.001	0.02 0.66	−0.17 0.002	− 0.32 < 0.001	− 0.17 0.002	− 0.20 < 0.001
**At discharge mRS**	**r** ***P*** **value**	0.27 < 0.001	0.24 < 0.001	0.00 0.90	− 0.09 0.08	− 0.08 0.12	− 0.12 0.02	−0.03 0.55

*PG-SGA* Patient-generated Subjective Global Assessment, *mRS* Modified Rankin Scale

Results of logistic regression analysis for evaluating the possible risk factors of mRS ≥ 3 (as outcomes), are presented in Table 5. Univariate analysis indicated significant associations between mRS ≥ 3 and age (OR: 1.02; 95%CI: 1.00–1.04), PG-SGA (OR: 1.08; 95%CI: 1.03–1.13), NIHSS (OR: 1.04; 95%CI: 1.02–1.07), dysphagia (OR: 1.69; 95%CI: 1.03–2.77), consciousness (OR: 1.48; 95%CI: 1.07–2.04), and mid-arm circumference (OR: 0.95; 95%CI: 0.90–1.00). These associations remained significant in multivariate analysis for PG-SGA (OR: 1.07; 95%CI: 1.00–1.13) and NIHSS (OR: 1.04; 95%CI: 1.01–1.07).

**Table 5:** Binary Logistic Regression With mRS Follow-Up as Output Variable

Univariate analysis				Multivariate analysis		
Variables	OR	95% CI	***P*** value	OR	95% CI	***P*** value
**Age (year)**	1.02	1.00–1.04	0.03	1.00	0.98–1.03	0.65
**BMI (kg/m**^**2**^**)**	0.96	0.90–1.01	0.13	NI	NI	NI
**PG-SGA**	1.08	1.03–1.13	0.001	1.07	1.00–1.13	0.02
**NIHSS**	1.04	1.02–1.07	< 0.001	1.04	1.01–1.07	0.001
**Dysphagia**	1.69	1.03–2.77	0.03	1.17	0.67–2.06	0.57
**Consciousness**	1.48	1.07–2.04	0.01	1.28	0.88–1.86	0.18
**Albumin (g/dl)**	1.00	0.84–1.20	0.93	NI	NI	NI
**Calf circumference (cm)**	0.96	0.91–1.01	0.18	NI	NI	NI
**Mid-arm circumference (cm)**	0.95	0.90–1.00	0.05	0.97	0.92–1.02	0.32
**Triceps skinfold (cm)**	0.96	0.93–1.00	0.07	NI	NI	NI

*OR* Odds ratio, *CI* Confidence interval, *NI* Not included, *mRS* Modified Rankin Scale, *PG-SGA* Patient-generated Subjective Global Assessment, *BMI* Body mass index, *NIHHS* National Institutes of Health Stroke Scale

**Fig. 1:**
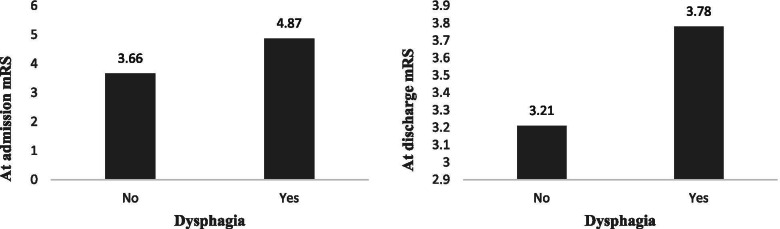
Difference in mRS score of patients with and without dysphagia, at the time of patients’ admission and discharge: Patients with dysphagia had significant higher mRS score at both admission and discharge time

Figure 1 demonstrates the mRS scores of participants with and without dysphagia at admission and discharge times. As is apparent, the mean mRS was significantly higher in patients with dysphagia at both admission and discharge times (*P* < 0.001).

## Discussion

To the best of our knowledge, the present study provided a clear relationship between clinical stroke outcomes and nutritional status. The lack of a standard and valid definition for malnutrition is a major problem in the assessment and evaluation of this condition. However, American Society for Parenteral and Enteral Nutrition (ASPEN) set out malnutrition as a subacute or chronic state of nutrition. The application of PG- SGA as a diagnostic measurement has been widely developed. PG-SGA has been used in older individuals and was created to consider medical issues and physical examination. Therefore, the methodology of the current study which have assessed the nutritional status of the participants strengthen the findings. Evidence indicates that few months after stroke, malnutrition leading to poor stroke outcome [9, 10]. Komaki et al. demonstrated that the frailty and fat mass measure as a method for evaluation of malnutrition may predict poor stroke related outcomes. Also, in younger groups, fat mass measure was a useful tool to predict poor outcomes in stroke patients [11]. In addition, previous studies illustrated that nutritional status at admission time may be associated with mortality and disability at discharge [11, 12]. In the current study, malnutrition is prevalent among stroke patients admitted to hospitals. According to our results, mal-nourished patients had a better condition regarding mRS at admission and discharge time, NIHSS, consciousness status, and hemoglobin compared to first quartile. Similarly, in Ramel’s study, anemic patients with lower hemoglobin level were also malnourished [13]. In contrast, malnourished patients (higher PG-SGA score) experienced lower levels of BMI, CC, TG, and hematocrit. As reported in the study of Ghorbani et al., severe malnourished patients showed a non-significant lower level of TG in comparison to well-nourished patients similarly [14]. In consistent with our findings, Wi et al. attempted to evaluate malnutrition among dialysis patients and demonstrated a significant difference between malnourished and non-malnourished individuals and lower level of TG, BMI and MAC and higher level of HDL among malnourished patients [15]. Also, in a study conducted by Nishiyama et al. malnourished patients presented lower values of hematocrit in comparison with well-nourished group [16].

Severe clinical outcomes of stroke along with malnutrition resulted in decreased level of BMI, CC and hematocrit. Zhang et al. acclaimed that malnutrition predicts poor stroke clinical outcomes which are consistent with our findings [17]. Malnutrition may affect inflammatory responses that influence recovery process in stroke patients [18, 19]. Therefore, this process highlights the vital role of nutritional intervention in promoting patient’s condition.

Consciousness status of patients correlated with mRS at admission and discharge times which is indicating that loss of consciousness happens as the stroke condition worsens. In addition, unpleasant nutrition status led to a decreased level of BMI, CC, MAC and TSF which negatively correlated with mRS at admission time. It has been shown that anthropometric indices including CC and MAC maybe a good predictors of diseases progression and mortality [20, 21].

Furthermore, discharge mRS negatively correlated with MAC. Nutritional status can affect anthropometric indices such as MAC. In another study conducted by Qiu et al., is reported that serum 25(OH) D levels was negatively associated with the risk of stroke recurrence [22]. Conflicting results have been reported in terms of different stroke related outcomes in underweight, normal weight, and overweight, or obese patients. In the study of Smith [19] and Ikenda [23], obese patients showed better clinical conditions. Whereas, in another study conducted by Dehlendorff et al., there was no significant differences regarding clinical outcomes of stroke among normal, overweight, and obese patients [24].

Moreover, albumin has been introduced as a marker for evaluating nutritional status and the previous studies tended to rely on serum albumin level as a measure of nutritional status but, it can be affected by disease too [25, 26]. Regarding to the findings of the current study, there was no significant difference about albumin level between PG-SGA quartiles. Also, no significant relationship was found between albumin and mRS at admission and discharge times. While, Gariballa et al. found that serum albumin was associated with mortality and poor outcomes in the acute stroke patients [9]. Similarly, Davalos et al. demonstrated an association between nutritional status, which have been evaluated with albumin level, and stroke severity [12]. Whereas, the study of Davis et al. failed to show any association between nutrition and stroke severity [9]. However, the albumin level was affected with nutritional status in the present study and malnourished stroke patients in the last quartile of PG-SGA experienced low and non-significant level of albumin compared to second quartile. The discrepancies in the results of previous studies which were conducted in this concept, may be attributed to different methodologies and statistics in relation to malnutrition prevalence, different study population, different values for malnutrition diagnosis, and nutritional assessment methods. Moreover, Nishiyama et al. designed a study to evaluate nutritional status of colorectal patients and acclaimed that malnourished patients have lower values of albumin in comparison with well-nourished group [16]. Some of the authors believe that lower serum albumin level (less than 4.0 to 4.5 g/dL) is associated with length of hospitalization and postoperative mortality [27, 28]. In the other hand, lower level of albumin in our study warn us about post recovery.

The present study had limitation that should be considered. We failed to assess our body composition of our included patients. Also, it seems that a longitudinal study of healthy individuals at risk of stroke may provide an accurate means in evaluating premorbid nutrition. However, as a strength, this study evaluated nutritional status using a validated measurement like PG-SGA. It seems that improving nutritional status may contribute to better functional outcomes among older stroke patients. Likewise, early identification of malnutrition should be encouraged as a part of medical care in stroke patients. In addition, better nutritional status may affect survival in people at risk of stroke. Therefore, it’s better to decrease the impact of malnutrition on stroke patients by improving nutrition strategies in stroke patients.

## Conclusion

This study revealed a direct and significant correlation between mRS at admission time and consciousness status, as well as negative correlations between mRS at admission time and BMI, CC, MAC, and TSF. In addition, at discharge time, there was positive correlations between mRS and consciousness status and PG-SGA score, as well as a negative one between mRS and MAC.

## Data Availability

The datasets generated and/or analyzed during the current study are not publicly available due for they are personal data but are available from the corresponding author on reasonable request.

## Abbreviations

PG-SGA: Patient-Generated Subjective Global Assessment
CT: Computed tomography
MRI: Magnetic resonance imaging
BMI: Body mass index
CC: Calf circumference
MAC: Mid-arm circumference
TSF: Triceps skinfold
TC: Total cholesterol
TG: Triglyceride
HDL-C: High-density lipoprotein cholesterol
LDL-C: Low-density lipoprotein cholesterol
hs-CRP: High-sensitivity C-reactive protein
ESR: Erythrocyte sedimentation rate
mRS: Modified Rankin Scale
NIHSS: National Institutes of Health Stroke Scale
